# Genetic diversity and genetic origin of Lanping black-boned sheep investigated by genome-wide single-nucleotide polymorphisms (SNPs)

**DOI:** 10.5194/aab-63-193-2020

**Published:** 2020-06-26

**Authors:** Heli Xiong, Xiaoming He, Jing Li, Xingneng Liu, Chaochao Peng, Dongmei Xi, Weidong Deng

**Affiliations:** 1 Yunnan Provincial Key Laboratory of Animal Nutrition and Feed, Faculty of Animal Science and Technology, Yunnan Agricultural University, Kunming 650201, People's Republic of China; 2 Yunnan Animal Science and Veterinary Institute, Kunming 650224, People's Republic of China; 3 Yunnan Kunming Police Dog Base of Ministry of Public Security, Kunming 650201, People's Republic of China

## Abstract

Lanping black-boned sheep was first discovered in the 1950s in Lanping county of China and characterized by black pigmentation on skin and internal organs. Due to the novel and unique trait, the genetic background of Lanping black-boned sheep is of great interest. Here, we genotyped genome-wide SNPs (single nucleotide polymorphisms) of Lanping black-boned sheep and Lanping normal sheep using Illumina OvineSNP50 BeadChip to investigate the genetic diversity and genetic origin of Lanping black-boned sheep. We also downloaded a subset SNP dataset of two Tibet-lineage sheep breeds and four other sheep breeds from the International Sheep Genomics Consortium (ISGC) as a reference for interpreting. Lanping black-boned sheep had a lower genetic diversity level when compared to seven other sheep breeds. Principal component analysis (PCA) showed that Lanping black-boned sheep and Lanping normal sheep were clustered into the Asian group, but there was no clear separation between the two breeds. Structure analysis demonstrated a high ancestry coefficient in Lanping black-boned sheep and Lanping normal sheep. However, the two populations were separated into two distinct branches in a neighbor-joining (NJ) tree. We further evaluated the genetic divergence using population $F_{\mathrm{ST}}$, which showed that the genetic differentiation that existed between Lanping black-boned sheep and Lanping normal sheep was higher than that between Tibet sheep and Changthangi sheep, which revealed that Lanping black-boned sheep is a different breed from Lanping normal sheep on the genetic level. In addition, structure analysis and NJ tree showed that Lanping black-boned sheep had a relatively close relation with Tibet sheep. The results reported herein are a first step toward understanding the genetic background of Lanping black-boned sheep, and it will provide informative knowledge on the unique genetic resource conservation and mechanism of novel breed formation.

## 1 Introduction

Lanping black-boned sheep (LPBB) was first discovered in the 1950s and characterized by black pigmentation on skin and internal organs compared to the reddish coloration in Lanping normal sheep (LPN) (Deng et al., 2008; Li et al., 2018); the pigmentation pattern is similar to that in silky fowl of China (Li et al., 2018; Deng et al., 2006). Attributable to their tender and tasty meat quality and no special smell of mutton, LPBB stands out among Lanping local sheep breeds and has been designated as a novel genetic resource by the Chinese Ministry of Agriculture (Li et al., 2018).

LPBB has the same morphology with LPN such as coat color and horns and tail shape; there are slight phenotypic differences between the two breeds seen by careful visible inspection: LPBB has dark teeth and gums and visible mucosa. However, there are great differences between the two breeds: the skin, muscle, and inner organs – including kidney, heart, lung, and trachea of LPBB – were dark colored compared to the red coloration in LPN (Deng et al., 2006). The husbandry system of LPBB is similar to semi-feral sheep breeds of Tibet sheep, which graze freely with more natural selection and less human intervention such as selective breeding and nutrition complements (Deng et al., 2006; Pan et al., 2018). Due to the phenotypic similarity with LPN and less human intervention, it is supposed by scientists that the formation of LPBB was the result of advantageous genetic mutations from LPN in the process of adaptation to the local harsh environments, which include high altitude (approximately 3000 m), strong radiation, steep terrain, and low temperatures during winter (Li, 2009). Meanwhile, some scholars believe that gene flow is present in Lanping local sheep and Tibet sheep because Lanping is located on the Tea Horse Road leading to Tibet in Yunnan and is adjacent to Diqing Tibetan Autonomous Prefecture (Li, 2009). Furthermore, previous studies reported that Yunnan-Kweichow Plateau sheep has closer relation with Qinghai-Tibetan Plateau sheep compared to northern Chinese sheep (Wei et al., 2015; Yang et al., 2016; Hu et al., 2019). Thus, investigating the genetic relation of LPBB with these sheep breeds may provide informative knowledge on genetic origin of LPBB.

Genetic diversity is an important population genetics parameter that helps to explain the process of evolution (Notter, 1999) and is a major concern, considering the necessity of conservation in local breeds (Meloni et al., 2015). The ability of a population to respond adaptively to environmental changes depends on its level of genetic diversity, and a species without enough genetic diversity is thought to be unable to cope with changing environments or evolving competitors and parasites (Khodabakhshzadeh et al., 2016). Lanping black-boned sheep was found initially with about 200 individuals and the population number increased slowly to 2000 from 2001 to 2005 (Deng et al., 2006). Due to the small population and isolated habitat, LPBB was expected to have low genetic diversity and increased incidence of inbreeding, which was not good for this unique and rare sheep breed. Thus, investigating genetic diversity of LPBB will provide a guide for the strategies on this genetic resource conservation.

To date, genes related to the mechanism of black pigmentation traits on LPBB have been studied such as *TYR* (Deng et al., 2008), *MC1R* (Deng et al., 2009a), *TYRP1, TYRP2* (Deng et al., 2009b), and *EDN3* (Darwish et al., 2018). However, the genetic background of LPBB has not yet been reported. In this study, we aimed to investigate the genetic diversity and genetic origin of Lanping black-boned sheep by genotyping LPBB and LPN using Illumina OvineSNP50 BeadChip. We also downloaded a subset of publicly available SNP (single-nucleotide polymorphism) BeadChip data (Kijas et al., 2012a) from the Ovine HapMap project of International Sheep Genomics Consortium (ISGC) (Kijas et al., 2012a) to use this established resource as a reference for interpreting genetic diversity and phylogenetic relationship within Lanping local breeds and for developing assumption about their relationship with worldwide breeds (Beynon et al., 2015). The results obtained in this study will be informative for future research and conservation of this unique breed.

## 2 Materials and methods

### 2.1 Animal materials

We collected 101 LPBB (48 males and 53 females) and 106 (19 males and 87 females) LPN sheep blood samples from Lanping County, Yunnan Province, China. Each individual was more than 1 year old and also carefully confirmed to match the breed standard. We then selected 15 LPBB (7 males and 8 females) and 15 LPN (7 males and 8 females) individuals randomly from the above collected blood samples to genotype; one LPN blood sample was lost for some reason. Genomic DNA was extracted from 500 µL of whole blood using Takara Blood Genome DNA Extraction Kit following the manufacture's guidelines.

All the experimental procedures were conducted under the International Animal Care and Use Committee of the Yunnan Agricultural University. The care and use of animals fully complied with local animal welfare laws, guidelines, and policies.

### 2.2 Genotyping and data quality control

In total, 29 genomic DNA samples were genotyped using Illumina OvineSNP50 BeadChip. The second dataset used in this study, including two Tibetan-lineage breeds (Tibetan and Changthangi sheep), another two Asian breeds (n=90), one southwest Asian (n=20) breed, and two southern and western European breeds (SW Europe) (n=43), was downloaded from ISGC and is available at http://www.sheephapmap.org (last access: 24 June 2020). Then, we merged and generated a 182-individual dataset (Table 1) with 49 034 overlapping SNPs for subsequent analysis. SNPs that cannot pass any of the following criteria were excluded from the analysis using PLINK 1.9 (Brito et al., 2017a; Johnston et al., 2016; Purcell et al., 2007): (1) with minor allele frequency (MAF) > 0.01; (2) with missing genotype data < 0.10; (3) individuals with missing genotype data  < 10 %; (4) with Hardy–Weinberg equilibrium P value > 0.00001; (5) included in the latest reference assembly of the sheep genome Oar_v4.1; and (6) located on autosomes. After filtering, a total of 45 754 autosomal SNPs were selected for further analyses. Individuals with potential kinships will also be excluded; we estimated the value of identity by state (IBS) between all samples using the “cluster – matrix” flag in PLINK to identify the genetic relatedness among sheep individuals (Gorkhali et al., 2016; Johnston et al., 2016).

**Table 1:** Summary of animal resources and genetic diversity of eight sheep populations.

Breed	Acronym	Origin	Number	*Pn*	*He*	*Ho*	F
Lanping black-boned	LPBB	Asian	14	0.8390	0.2861	0.3027	0.1847
Lanping normal	LPN	Asian	15	0.8590	0.2870	0.2889	0.2290
Tibet	TIB	Asian	37	0.9551	0.3390	0.3209	0.1392
Changthangi	CHA	Asian	29	0.9682	0.3491	0.3345	0.1171
Bangladeshi	BGE	Asian	24	0.8856	0.3079	0.2691	0.2835
Norduz	NDZ	Southwest Asian	20	0.9355	0.3309	0.3549	0.0623
Chinese Merino	CME	SW Europe	23	0.9740	0.3551	0.3688	0.0267
Sardinian Ancestral Black	SAB	SW Europe	20	0.9497	0.3332	0.3560	0.0535

*Pn* represents the proportion of SNP displaying polymorphism, *He* represents average expected heterozygosity, *Ho* represents average observed heterozygosity, and F represents inbreeding coefficient.

### 2.3 Genetic diversity

Five metrics were used to evaluate levels of within-breed genetic diversity, and the values were estimated using PLINK 1.9 (Purcell et al., 2007). The proportion of polymorphic SNP (*Pn*) gives the fraction of total SNP that displayed both alleles within each population (Brito et al., 2017a). *Pn* was calculated as the proportion of SNPs with average minor allele frequency (MAF) greater than 1 % within each breed (Brito et al., 2017a). MAF is the frequency estimate of the least common allele per breed and estimated using the “freq” flag. Expected heterozygosity (*He*) and observed heterozygosity (*Ho*) were estimated using the “hardy” flag, and inbreeding coefficient (F) was estimated using the “het” flag (Beynon et al., 2015; Brito et al., 2017a).

### 2.4 Phylogenetic analysis

A pruned dataset of 182 sheep containing 36 711 SNPs, which excluded SNPs in LD (linkage disequilibrium) (PLINK – indep-pairwise 50 10 0.2; – extract LD0.2.prune.in), was used to investigate the genetic structure. Principal component analysis (PCA) was performed with GCTA software (version 1.26.0, genome-wide complex trait analysis) (Yang et al., 2011). Population structure was evaluated using Structure software (version 2.3.4) (Falush et al., 2007). The neighbor-joining (NJ) tree was constructed using MEGA software (version 7.0) on the basis of all SNPs (Hall, 2013). The pairwise $r^{2}$ value within each population was calculated with parameter “– r2 – ld-window 99999 – ld-window-r2 0.2” in PLINK to compare LD patterns among breeds. Population divergence was calculated as $F_{\mathrm{ST}}$ using VCFtools (version 4.2) (Danecek et al., 2011).

## 3 Results

### 3.1 Genetic diversity

**Figure 1:**
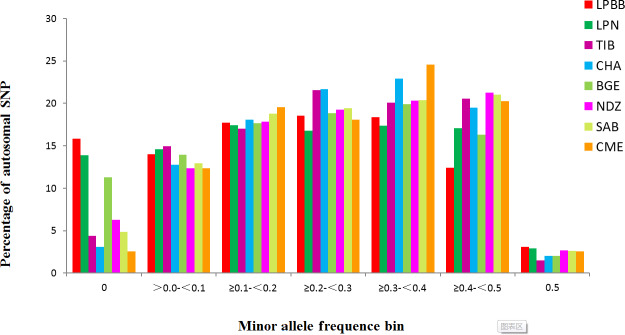
Minor allele frequency (MAF) of eight sheep breeds.

Inbred individuals were not observed in the 182 studied sheep according to the IBS score (IBS < 0.9) (Yang et al., 2016). The level of *Pn* present in LPBB population was 0.8390, which was slightly lower than LPN population but greatly lower than the other six populations; this reflected that LPBB population had lower loci displaying polymorphism compared to the remaining populations (Table 1). The distribution of MAF per breed is given in Fig. 1 and shows that the LPBB population has the lowest level of highly variable SNPs (MAF > 0.3) with a proportion of 33.88 %, and the highest level is CME with a proportion of 47.41 %; LPBB population had an excess of low allele frequency SNPs (MAF = 0) compared with the other breeds. The value of *He* was close to *Ho* in all populations and the LPBB population had the lowest *He* (0.2861), followed by LPN population (0.2870); compared to pure breeds, the cross breed of CME had the highest level of *He* (0.3551). The inbreeding coefficients in all populations were detected with weak to moderate levels, ranging from 0.0267 to 0.2835 and the level in the LPBB population was moderate (0.1847).

### 3.2 Phylogenetic analyses

**Figure 2:**
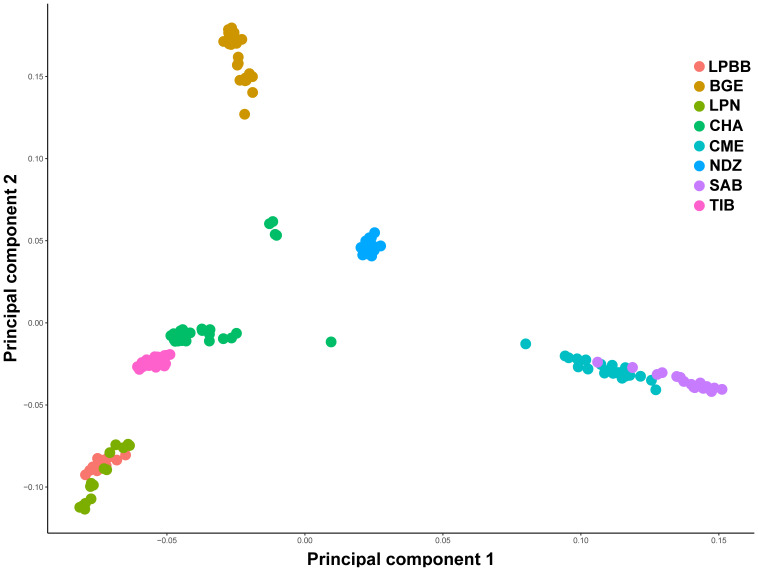
Principal components 1 and 2 for the 182 sheep individuals.

We first performed principle component analysis (PCA) based on the pruned genotype data of 36 711 SNPs among all individuals to examine the genetic relationship between LPBB and its geographic neighbors and worldwide sheep breeds. The largest PC (6.931 % of total variation) separated eight breeds into three groups consistent with their genetic origin which were Asian breeds (PC1 < 0, LPBB, LPN, TIB, CHA and BGE), southwest Asian (0 < PC1 < 0.05, NDZ), and SW European breeds (PC1 > 0.05, CME, SAB). The second PC (4.467 % of total variation) separated Asian sheep breeds into three clusters consistent with their geographical distance: Lanping local breeds (PC2 < -0.05, LPBB and LPN), Tibet-lineage breeds (-0.05 < PC2 < 0.05, TIB and CHA), and BGE, which is an Asian breed far away from the first two groups (PC2 > 0.05). Furthermore, LPBB and LPN populations showed relatively close relation with TIB and CHA populations. Based on PC1 and PC2, LPBB individuals clustered more tightly than LPN individuals; however, there was not a clear separation between LPBB and LPN populations. In addition, five individuals of CHA were outside of their expected population clusters (Fig. 2).

**Figure 3:**
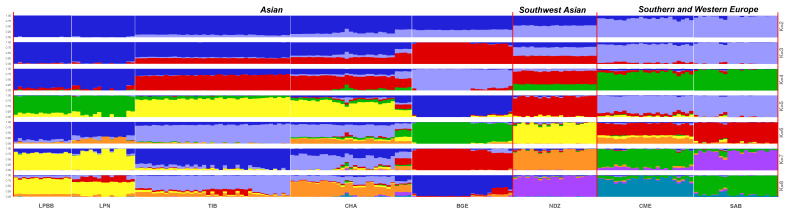
Population structure of 182 sheep individuals inferred from Structure 2.3.4 software. The length of each colored segment represents the proportion of the individual genome inferred from ancestral populations (K=2–8).

**Figure 4:**
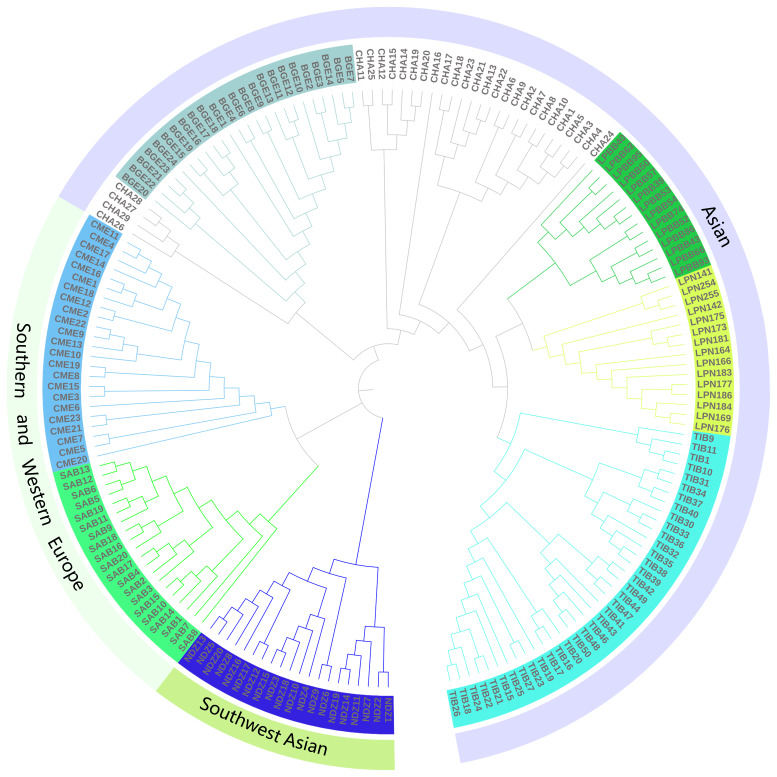
The NJ tree was constructed using MEGA 7.0 and edited by iTOL.

**Figure 5:**
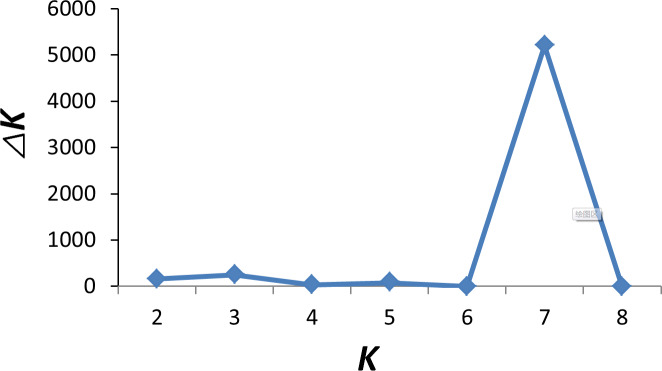
Optimal value of K determined by δK (ΔK).

To investigate ancestry and admixture proportion of LPBB breed, we performed population structure analysis based on pruned SNPs. Model-based clustering of the individuals was analyzed by assuming numbers of populations (K) between 1 and 8 (Beynon et al., 2015). Figure 3 shows the distribution of individuals into clusters for K=2–8. The clustering analysis for K=2 shows that 182 sheep were genetically divided into Asian and SW European breeds; NDZ population had an admixture component of Asian breeds and SW European breeds. When K=3, BGE breed was separated from Asian breeds, which was consistent with the geographical origin and the PCA analysis that PC2 separated BGE breeds from the remaining Asian breeds. When the K value became large, most breeds tended to be separated except LPBB and LPN. The ideal clustering of eight populations determined by the ΔK method (Nie et al., 2016) (Fig. 5) was K=7. LPBB and LPN breeds were clustered into one population at K=7; this revealed that LPBB and LPN breeds had a very similar genetic background, which was consistent with the PCA that LPBB and LPN were mixed. Figure 4 shows that at K=7 LPBB and LPN sheep are represented by one main cluster and have some features that are present in the TIB cluster. Although LPBB and LPN were clustered together, there are differences between the two populations: the admixture level of LPBB was slightly higher than that of LPN, LPBB had more features that presented in TIB clusters than LPN the representative clusters in CHA were present in LPN with a low ancestry coefficient. Meanwhile, a similar pattern was observed in TIB and CHA clusters; the representative clusters in LPBB and LPN were also present in TIB and CHA clusters with a low ancestry coefficient. This result demonstrated that LPBB and LPN breeds had shared ancestry with TIB breed or there was a gene flow between them.

To further resolve the phylogenetic relationship of LPBB with their geographical neighbors and worldwide sheep breeds, we constructed a neighbor-joining tree based on the whole genome-wide SNPs after filtering. Agreeing with PCA analysis, the phylogenetic tree split all breeds into three distinct branches which represent Asian, southwest Asian, and SW European sheep breeds (Fig. 3) and five individuals of CHA were outside of their clusters. The phylogenetic tree shows that LPBB and LPN breeds are separated into two clear clusters and positioned in one clade with TIB and CHA breed. Furthermore, LPBB and LPN breeds had the shortest branch length compared to the TIB breed, which revealed that LPBB and LPN had a relatively close relationship with TIB. The fact that they had common ancestry or gene flow between them was confirmed by the structure analysis.

Due to the morphology and genetic similarity of LPBB and LPN breeds, it was questionable that LPBB and LPN were distinct enough to be considered a different breed. To investigate their genetic difference, we used populations $F_{\mathrm{ST}}$ to evaluate the degree of genetic divergence between LPBB and LPN sheep breeds and compared them against the divergence that exists between populations recognized as separate breeds. The pairwise population $F_{\mathrm{ST}}$ of eight breeds was calculated using VCFtools. The $F_{\mathrm{ST}}$ value can range from zero (no genetic differentiation between the populations or from the ancestral population) to one (fully differentiated in each population). $F_{\mathrm{ST}}$ values ranging from 0.05 to 0.15 and 0.15 to 0.25 mean moderate and large differentiations, respectively (Grasso et al., 2014). Table 2 shows the $F_{\mathrm{ST}}$ that existed between each breed pair. The $F_{\mathrm{ST}}$ value of all pairwise was small to moderate, which was consistent with the report by Kijas et al. (2009). They studied 74 breeds from different parts of the world and showed that sheep breeds had maintained generally low genetic differentiation. The lowest level of divergence was found between TIB and CHA breeds (0.0285), which are both Tibet-lineage sheep breeds, while the greatest was observed between LPBB and SAB (0.1584), which are Asian and SW European breeds, respectively; this result was supported by the structure analysis result that they were from completely distinct clusters. Moderate genetic divergence was observed (0.0646) between LPBB and LPN breeds, which was higher than that between the two Tibet-lineage separate breeds. This strongly indicated that LPBB breed is sufficiently different with the LPN breed and can be considered a separate breed. The divergence level of LPBB-TIB and LPN-TIB was relatively lower with $F_{\mathrm{ST}}$ values of 0.0750 and 0.0759, respectively, which agreed with the NJ tree analysis that LPBB and LPN were more related to TIB than to the remaining breeds.

**Table 2:** Population divergence measured as $F_{\mathrm{ST}}$.

		Pairwise population divergence measured as $F_{\mathrm{ST}}$ (SD)${}^{*}$							
Breed	Acronym	LPBB	LPN	TIB	CHA	BGE	NDZ	CME	SAB
Lanping black-boned	LPBB		0.0646	0.0750	0.0765	0.1397	0.1291	0.1353	0.1584
Lanping normal	LPN	0.0073		0.0759	0.0776	0.1389	0.1298	0.1339	0.1566
Tibet	TIB	0.0056	0.0059		0.0285	0.0962	0.0873	0.1050	0.1251
Changthangi	CHA	0.0069	0.0062	0.0024		0.0815	0.0744	0.0900	0.1114
Bangladeshi	BGE	0.0102	0.0102	0.0054	0.0066		0.1140	0.1316	0.1501
Norduz	NDZ	0.0125	0.0113	0.0077	0.0079	0.0104		0.1019	0.1221
Chinese Merino	CME	0.0143	0.0123	0.0088	0.0087	0.0099	0.0092		0.0872
Sardinian Ancestral Black	SAB	0.0164	0.0152	0.0135	0.0138	0.0163	0.0161	0.0210	

${}^{*}$ $F_{\mathrm{ST}}$ is given above the diagonal and its standard deviation (SD) for each combination is given below.

**Figure 6:**
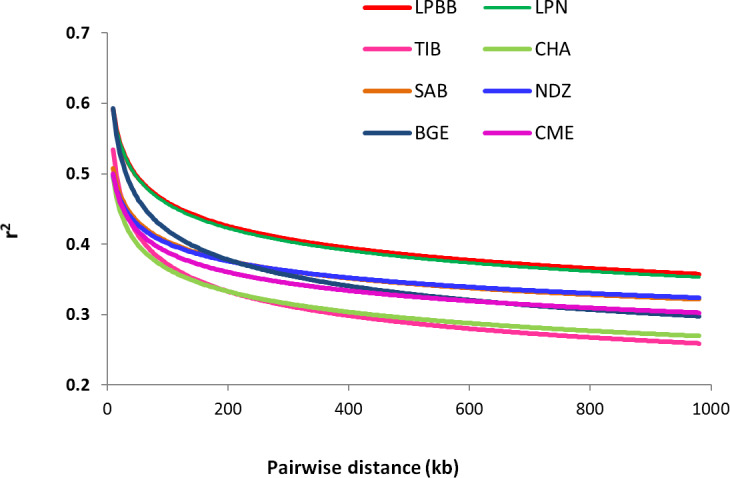
Linkage disequilibrium (LD) decay of eight sheep breeds, with one line per breed.

To better understand the population variation, we performed linkage disequilibrium (LD) decay analysis, which can be informative for population demography and assessing the number of markers required to associate genetic variation with traits. Estimates of LD based on the $r^{2}$ value were different between the eight populations. Compared to other breeds, LPBB population showed an overall slow decay rate and a high level of LD, which suggested that it was derived from a relatively small ancestral population (Fig. 6). Furthermore, the high LD level of LPBB population shows that there is strong linkage between SNP markers, and a lower marker density was required in the genome-wide association study.

## 4 Discussion

In this study, we evaluated for the first time the genetic diversity and population structure of LPBB with seven other populations. The mean values of the three metrics (*Pn, He*, F) of the downloaded six populations in this study were slightly different but had the same trend with the values reported in Kijas et al. (2012a); this might be the result of different filter criteria of SNPs.

Analysis of genetic diversity revealed that the LPBB population had the lowest level of diversity with an estimated genetic diversity (*He*) of 0.2861 and polymorphic SNPs (*Pn*) of 0.8390 (Table 1) when compared to other breeds. The lower level of genetic diversity of LPBB population was also detected in the distribution of MAF and confirmed by the lower rate of LD decay in LPBB. CME, a crossbreed of Australian Merino × Xinjiang sheep, had the highest level of diversity with an estimated *He* of 0.3551 and *Pn* of 0.9740; this was consistent with the report that the crossbreed has a higher genetic diversity level compared to the pure breed (Meadows et al., 2008; Brito et al., 2017b). Furthermore, Australian Merinos were reported as the most diverse sheep population (Meadows et al., 2008). The low genetic diversity in the LPBB population can be explained by their geographically separated habitat and small population size (Meloni et al., 2015). Lanping black-boned sheep have distributes in three towns of Yuping Mountain of Lanping. There was dozens of Lanping black-boned sheep individuals when first discovered in the 1950s, and until 2000 their number increased slowly to several hundred. In addition, due to the high elevation and steep road of Yuping Mountain, there is a geographically isolated area, and no sheep genes were introduced. The higher LD level and slow decay of LPBB population also demonstrated that the population size was relatively small. Genetic diversity is correlated with fitness (Silva et al., 2006): populations with low level of genetic diversity are expected to be less able to adapt to environmental changes (Meloni et al., 2015; Carrol and Fox, 2008) or evolving competitors and parasites (Khodabakhshzadeh et al., 2016); thus, it is suggested to conserve this unique breed.

Population structure was analyzed using PCA, structure, and NJ tree. The PCA analysis showed that the LPBB population was clustered into Asian group and mixed with LPN population, which was confirmed by the structure analysis that LPBB individuals were grouped into Asian population when K=2 and LPBB and LPN individuals were clustered into one population at the optimal K value (K=7). This result revealed that LPBB and LPN populations had great genetic similarity, which strongly explained the morphology similarity between LPBB and LPN individuals. This raises questions about whether the two populations are sufficiently different to be considered separate breeds. To search for evidence of genetic divergence, we used population $F_{\mathrm{ST}}$ to estimate genetic differentiation between the two populations and other breeds. The genetic differentiation observed between LPBB and LPN ($F_{\mathrm{ST}}=0.0646$) was higher than that between the recognized separate breeds of TIB and CHA ($F_{\mathrm{ST}}=0.0285$), which were both Tibet lineage. In addition, Kijas et al. (2012b) choose a subset of genotyped data from ISGC and grouped them into selection lines within breed, breed pairs of Mediterranean origin, and breed pairs of southern vs. northern European origin. $F_{\mathrm{ST}}$ values of these three groups were 0.017, 0.042, and 0.114, respectively, and this showed that the $F_{\mathrm{ST}}$ value that existed between LPBB and LPN was higher than selection lines within breed and breed pairs of Mediterranean origin. Therefore, on the basis of genetic data, we can conclude that LPBB can be considered a different breed from LPN.

LPBB population was clustered into Asian group and mixed with LPN population in both PCA and structure analysis, which demonstrated that LPBB has an Asian origin and had a very close relation with the LPN breed. Furthermore, the structure analysis revealed that LPBB and LPN breeds had shared ancestry with TIB breeds, which coincided with the NJ tree analysis that LPBB and LPN individuals were clustered into one clade with TIB individuals. This result agreed with the traditional classification that Lanping local sheep were Tibet lineage due to their phenotype traits such as thin tail (Hu et al., 2019) and carpet wool (Wei et al., 2015). To date, there are more than 42 native sheep breeds established in China, and they are classified into three major lineages by their geographic distribution known as northern China, Qinghai-Tibetan Plateau, and Yunnan-Kweichow Plateau sheep (Hu et al., 2019; Yang et al., 2016); Yunnan-Kweichow Plateau sheep had closer affinity with Qinghai-Tibet sheep compared with northern China sheep and could be explained by their thin-tail origin (Hu et al., 2019). Lanping is located on the Tea Horse Road leading to Tibet in Yunnan and is adjacent to Diqing Tibetan Autonomous Prefecture. This study provided the genetic evidence that LPBB sheep had close affinity with Tibet sheep. For a more accurate understanding of the origin of LPBB sheep breeds, the relation with other Yunnan-Kweichow Plateau breeds should be studied.

## 5 Conclusions

In this study, we estimated for the first time the genetic diversity and genetic origin of Lanping black-boned sheep by using genome-wide SNP data. We observed that the LPBB population had the lowest genetic diversity compared to its geographical neighbors and four other world breeds. This finding improved our understanding of the genetic diversity in LPBB sheep breed and suggested that strategies should be implemented to maintain or increase genetic diversity in this breed. The PCA and structure analysis revealed that LPBB and LPN populations had great genetic similarity, whilst the NJ tree and genetic differentiation showed that there are differences between LPBB and LPN populations, and LPBB can be considered a specific separate breed. In addition, this study demonstrated that LPBB and LPN populations had common ancestry with the TIB population, which provided insight into the genetic origin of LPBB sheep and will be useful for the novel breed formation research.

## Data Availability

The genotyped data reported in this article are available upon request for research purposes.
